# Cytoplasmic Retention of a Nucleocytoplasmic Protein TBC1D3 by Microtubule Network Is Required for Enhanced EGFR Signaling

**DOI:** 10.1371/journal.pone.0094134

**Published:** 2014-04-08

**Authors:** Ze He, Tian Tian, Dan Guo, Huijuan Wu, Yang Chen, Yongchen Zhang, Qing Wan, Huzi Zhao, Congyang Wang, Hongjing Shen, Lei Zhao, Xiaodong Bu, Meiling Wan, Chuanlu Shen

**Affiliations:** 1 Department of Pathology and Pathophysiology, Medical School, Southeast University, Nanjing, Jiangsu, People's Republic of China; 2 Key Laboratory of Developmental Genes and Human Diseases, Ministry of Education, Institute of Life Sciences, Southeast University, Nanjing, Jiangsu, People's Republic of China; 3 Department of Biomedical Sciences, University at Buffalo, State University of New York, Buffalo, New York, United States of America; University of Luebeck, Germany

## Abstract

The hominoid oncogene TBC1D3 enhances epidermal growth factor receptor (EGFR) signaling and induces cell transformation. However, little is known regarding its spatio-temporal regulation and mechanism of tumorigenesis. In the current study, we identified the microtubule subunit β-tubulin as a potential interaction partner for TBC1D3 using affinity purification combined with mass spectrometry analysis. The interaction between TBC1D3 and β-tubulin was confirmed by co-immunoprecipitation. Using the same method, we also revealed that TBC1D3 co-precipitated with endogenous α-tubulin, another subunit of the microtubule. In agreement with these results, microtubule cosedimentation assays showed that TBC1D3 associated with the microtubule network. The β-tubulin-interacting site of TBC1D3 was mapped to amino acids 286∼353 near the C-terminus of the TBC domain. Deletion mutation within these amino acids was shown to abolish the interaction of TBC1D3 with β-tubulin. Interestingly, the deletion mutation caused a complete loss of TBC1D3 from the cytoplasmic filamentous and punctate structures, and TBC1D3 instead appeared in the nucleus. Consistent with this, wild-type TBC1D3 exhibited the same nucleocytoplasmic distribution in cells treated with the microtubule depolymerizing agent nocodazole, suggesting that the microtubule network associates with and retains TBC1D3 in the cytoplasm. We further found that deficiency in β-tubulin-interacting resulted in TBC1D3's inability to inhibit c-Cbl recruitment and EGFR ubiquitination, ultimately leading to dysregulation of EGFR degradation and signaling. Taken together, these studies indicate a novel model by which the microtubule network regulates EGFR stability and signaling through tubulin dimer/oligomer interaction with the nucleocytoplasmic protein TBC1D3.

## Introduction

The epidermal growth factor receptor (EGFR) is the first identified member of the ErbB receptor tyrosine kinase family. The receptor activates a wide variety of signaling pathways, with the Ras-ERK pathway as perhaps the best characterized of these pathways. EGFR signaling controls numerous critical cellular processes, such as cell survival, proliferation, differentiation and locomotion [1], [2]. After activation, the receptor must be inactivated to prevent prolonged stimulation of cells via feedback control mechanisms, including activation of phosphatases, post-translational modifications and endocytosis of the receptor. Excessive activation of EGFR has been associated with the development and progression of numerous tumors [3].

As one of the most common post-translational modifications, ubiquitination of EGFR plays a critical role in endocytic trafficking and lysosomal degradation of the activated receptor. Cbl is a RING domain E3 ubiquitin ligase responsible for EGFR ubiquitination [4], [5]. There appears to be two distinct mechanisms underlying the ubiquitination of EGFR by Cbl. The first one is mediated by the C-terminal phosphorylated tyrosine residue Tyr 1045 of EGFR, which directly binds to the N-terminal tyrosine kinase binding domain of Cbl [6], [7]. The alternative mechanism involves the tyrosine residues Tyr 1068 and Tyr 1086 of the activated receptor, which indirectly recruits Cbl through its interaction with the SH3 domain of Grb2 [8]. With Cbl serving as an adaptor to bridge Ubc4/5 E2 ubiquitin-conjugating enzyme interaction with EGFR, ubiquitin is transferred directly from the E2 to distinct lysine residues within the kinase domain of EGFR, including six major ubiquitin conjugation sites (Lys692, Lys713, Lys, 730, Lys843, Lys905, and Lys946) [9], [10]. Although sufficient but not essential for EGFR internalization [11]–[13], EGFR ubiquitination is indeed required for its sorting onto intraluminal vesicles of multivesicular endosomes/bodies and subsequent lysosomes for efficient degradation [12], [14].

Diverse negative or positive regulators of EGFR ubiquitination have been identified, including Sprouty2, Cdc42, intersectin-1, protein-tyrosine kinase 6 (PTK6) and TBC1D3. Among them, phosphorylated Sprouty2 and activated Cdc42, in complex with the Cbl-interacting protein of 85 kDa and Cool-1, respectively, negatively regulates the ubiquitination of EGFR through sequestration of Cbl away from the activated receptor [15]–[17]. Conversely, the multi-domain scaffolding protein intersectin-1 stimulates its ubiquitination through competitively inhibiting Sprouty2 from binding to Cbl [18]. In contrast to association with Cbl, PTK6, a non-receptor protein-tyrosine kinase competes with Cbl for binding to the phosphorylated Y1045 on EGFR, leading to disruption of EGFR-Cbl interaction and inhibition of EGFR ubiquitination [19].

TBC1D3 (also called prostate cancer gene 17, PRC17) is a hominoid-specific gene that was originally identified as a novel amplified oncogene, based on its ability to confer tumorigenicity to NIH 3T3 cells [20]–[22]. The oncogene is amplified in 15% of primary prostate tumors and in approximately 50% of metastatic prostate tumors [22]. Consistent with many other hominoid-specific genes, TBC1D3 underwent several segmental duplications during primate evolution, resulting in the existence of eight paralogues organized in two clusters within the 17q12 genomic region [23]. TBC1D3 possesses an N-terminal TBC (Tre-2, Bub2, Cdc16) domain, which generally encodes GTPase-activating proteins (GAPs) for Rab family GTPases [24]. However, no GAP activity could be detected because the TBC domain of TBC1D3 lacks the conserved arginine and glutamine residues essential for catalytic activity [25]. Instead, TBC1D3 promotes EGF and insulin signaling by inhibiting or substantially delaying ubiquitination and subsequent degradation of EGFR and insulin receptor substrate-1 (IRS-1), respectively, thereby triggering cell proliferation [26], [27]. The delay in IRS-1 ubiquitination has been linked to TBC1D3-induced activation of protein phosphatase 2A (PP2A). Following the activation, PP2A dephosphorylates and inactivates p70 S6 kinase (S6K), leading to suppression of phosphorylation at key sites required for IRS-1 binding to Cul7 E3 ubiquitin ligase [27]. Similarly, TBC1D3 reduces EGFR ubiquitination through suppression of Cbl recruitment and binding to the receptor [26]. Most recently, TBC1D3 was shown to be ubiquitinated and degraded by the Cul7 E3 ligase in response to serum stimulation [28]. However, little is known regarding the mechanism by which TBC1D3 inhibits the recruitment of Cbl. Furthermore, spatio-temporal regulation of TBC1D3 remains undefined.

In the current study, we report that tubulin/microtubule interacts with TBC1D3. We further identify TBC1D3 as a nucleocytoplasmic protein and its association with tubulin/microtubule regulates its nucleocytoplasmic distribution. Finally, our work reveals a novel model by which microtubule network regulates EGFR stability and signaling through tubulin dimer/oligomer interaction with TBC1D3.

## Materials and Methods

### Antibodies

For immunofluorescence of β-tubulin, anti-β-tubulin antibody (E021040, Earthox) was used; for immunoprecipitation, anti-HA (sc-805, Santa Cruz) and anti-β-tubulin (#21335, Signalway Antibody) antibodies were used. Anti-EGFR antibody (#21074, Signalway Antibody) was used for both immunofluorescence and immunoprecipitation. For immunoblotting, antibodies against EGFR (#4267), HA (#2367), p44/42 MAPK (#9107), and phospho-p44/42 MAPK (#9106) were purchased from Cell Signaling Technology, antibody against c-Cbl (#21549) from Signalway Antibody Co., Ltd., antibodies against α-tublin (T9026) and acetylated α-tublin (T7451) from Sigma, antibodies against GAPDH (AP0063) and HDAC6 (BS1165) from Bioworld Technology, Inc. and anti-ubiquitin (Ub) antibody (FK2) from Enzo Life Sciences.

The secondary antibodies used were Rhodamine (TRITC)-conjugated goat anti-mouse immunoglobulin G (IgG; heavy and light chain; Jackson Immunoresearch Laboratories) and DyLight 594 AffiniPure goat anti-rabbit immunoglobulin G (IgG; heavy and light chain; EarthOx, LLC).

### Plasmids

Human TBC1D3 and GGA3 cDNAs were amplified by reverse transcription-PCR from total RNA using the following primers:

TBC1D3 forward primer, 5′-TAGGATCCATGGACGTGGTAGAGGTCGC-3′;

TBC1D3 reverse primer, 5′-GCGAATTCTAGAAGCCTGGAGGGAACTG-3′;

GGA3 forward primer, 5′-GAGAATTCATGGCGGAGGCGGAAGG-3′;

GGA3 reverse primer, 5′-CTCTCGAGTCATAGGTTCCCCCACTGTTC-3′.

After their sequences were confirmed by direct automated DNA sequencing, the entire open reading frame (ORF) of TBC1D3 was inserted into *Bam*H I/*Eco*R I site of HA-pcDNA3.0 and pEBG vectors to generate HA- and GST-tagged fusion proteins (HA-TBC1D3 and GST-TBC1D3), respectively. The entire ORF of GGA3 was ligated into *Eco*R I/*Xho* I site of pGEX-6P-1 vector to generate a GST-tagged fusion protein (GST-GGA3). pEGFP-TBC1D3 encodes a EGFP-tagged TBC1D3 protein (EGFP-TBC1D3) and was generated by PCR. HA-TBC1D3(Δ252-285)/pcDNA3, HA-TBC1D3(Δ286-319)/pcDNA3, HA-TBC1D3(Δ320-353)/pcDNA3, and pEGFP-TBC1D3(Δ320-353), harboring internal deletions of the indicated residues, were generated by overlap extension PCR. HA-TBC1D3(251)/pcDNA3, HA-TBC1D3(353)/pcDNA3, HA-TBC1D3(476)/pcDNA3, pEGFP-TBC1D3(251), pEGFP-TBC1D3(353), pEGFP-TBC1D3(476) and pGEX-GGA3(298) encode the indicated N-terminal residues of the corresponding genes and were generated by PCR. Further details are available upon request.

### Cell Culture and Transfections

Human SMMC7721 hepatocellular carcinoma cells were obtained from the Committee on Type Culture Collection of Chinese Academy of Sciences (Shanghai, China) and grown in Dulbecco's Modified Eagle's Medium (DMEM, Invitrogen) containing 2 mM of glutamine and supplemented with 10% heat-inactivated fetal bovine serum, 100 U/ml of penicillin and 100 μg/ml of streptomycin at 37°C in 5% CO2. SMMC7721 cells were transfected with LipoD293 Reagent (SignaGen) according to manufacturer's instructions. Approximately 24 h after transfection, cells were processed as described for each experiment.

### Signaling, EGFR Degradation and Western Blot Assays

Erk1/2 activation and EGFR degradation were measured in cells serum-starved for 24 h. Cells were incubated in the presence of 100 ng/ml EGF (PeproTech) at 37°C for different time points, washed with ice-cold phosphate-buffered saline (PBS), and lysed in RIPA buffer (50 mM Tris, pH 7.5, 150 mM NaCl, 0.1% SDS, 0.5% sodium deoxycholate, 1% TritonX-100, 1 mM phenylmethylsulfonyl fluoride, 5 μg/ml leupeptin, 2 μg/ml aprotinin, 5 μg/ml pepstatin, 1 mM sodium orthovanadate, 10 mM sodium fluoride). The lysates were clarified by centrifugation, and protein concentrations were determined using the Bradford assay. Proteins were separated by SDS PAGE and electroblotted onto a 0.45-μm pore PVDF membrane, which was blocked in 2% bovine serum albumin in TBST buffer (25 mM Tris [pH 7.4], 150 mM NaCl, 0.05% Tween 20), probed with anti-EGFR, anti-p44/42 MAPK, anti-phospho-p44/42 MAPK, anti-HA and anti-α-tubulin antibodies, washed with TBST and incubated with horseradish peroxidase-conjugated goat anti-rabbit IgG (Santa Cruz) or goat anti-mouse IgG (Bioworld) for detection by SuperSignal West Pico Chemiluminescent Substrate (Pierce). Immunoblotting data were quantified by Image-Pro Plus 6.0 software (Media Cybernetics, Inc.).

### GST Pull-down and Co-immunoprecipitation

Cells were lysed in ice-cold lysis buffer (20 mM Tris-Cl, pH 7.5, 0.5% Triton X-100, 150 mM NaCl, 1 mM EDTA, 1 mM EGTA, 10 mM sodium fluoride, 1 mM sodium orthovanadate, 1 mM phenylmethylsulfonyl fluoride, 5 μg/ml pepstatin, 5 μg/ml leupeptin, 2 μg/ml aprotinin). For analysis of EGFR ubiquitination, additional 10 mM N-ethylmaleimide was added into lysis buffer. After incubation on ice for 15 min, the lysates were centrifuged at 13,000 rpm for 10 min at 4°C. For GST pull-down, an aliquot of the clarified supernatant was removed for direct immunoblotting, and the remainder was incubated with GST, GST-TBC1D3, or GST-GGA3(298) fusion proteins attached to the glutathione-Sepharose beads (Amersham) for 4 h at 4°C with constant mixing. After centrifugation, the beads were washed three times in the lysis buffer. Samples were boiled, fractionated by SDS PAGE, stained with 1% Coomassie brilliant blue or Ponceau S followed by immunoblotting with anti-HA antibody, and then detected by enhanced chemiluminescence (Pierce). For co-immunoprecipitation, the clarified lysates were incubated with the indicated antibodies for 1 h and then with Protein G PLUS-Agarose beads (Santa Cruz) for 4 h or overnight at 4°C. Beads were washed three times in the lysis buffer. Samples were separated by SDS PAGE and then immunoblotted with the indicated antibodies.

### Peptide Mass Fingerprinting Analysis

SMMC7721 cells were transfected with GST-TBC1D3 or control GST. The following day, cell extracts were pulled down (PD) with glutathione-Sepharose 4B beads. After SDS PAGE, the gels were stained with Coomassie brilliant blue. The differentially bound protein bands were then excised from the SDS PAGE gel, in-gel digested by trypsin, and analyzed by Shanghai GeneCore BioTechnologies Co., Ltd. using the Proteomics Solution 1 System (Applied Biosystems Co., Ltd., USA). To identify the proteins, peptide masses from MALDI-TOF MS were matched with the theoretical molecular weight of peptides for proteins in the NCBI database using MASCOT search tools (www.matrixscience.com).

### Microtubule Cosedimentation Assays

After transfected with HA-TBC1D3, SMMC7721 cells were scraped in 0.5 ml of General Tubulin Buffer (80 mM PIPES, pH 7.0, 2 mM MgCl_2_, 1 mM EGTA) supplemented with 1 mM GTP, 1 mM phenylmethylsulfonyl fluoride, 5 μg/ml leupeptin, 2 μg/ml aprotinin, 5 μg/ml pepstatin, 1 mM sodium orthovanadate, 10 mM sodium fluoride, collected and then sonicated 5 times at 4°C for 15 seconds with a 1 minute cooling period between each burst. The lysates were centrifuged at 100,000×*g* for 40 min at 4°C to remove any microtubules. Clarified lysates were then used for microtubule binding protein spin-down assays using a commercially available kit (Cytoskeleton Inc. Denver, CO, cat.# BK029). Briefly, 2 μl of Cushion Buffer (80 mM PIPES, pH 7.0, 1 mM MgCl_2_, 1 mM EGTA, 60% glycerol) was added to 20 μl aliquots of tubulin (100 μg of protein dissolved in General Tubulin Buffer containing 1 mM GTP) and incubated at 35°C for 20 min. Newly formed microtubules were stabilized by the addition of 200 μl of General Tubulin Buffer containing 20 μM Taxol. Clarified lysates were then added to the Taxol supplemented General Tubulin Buffer alone (MTs(-)) or together with polymerized microtubules (MTs(+)). The microtubule binding protein fraction (MAPF, containing MAP2 and tau proteins) and BSA were used as positive and negative controls, respectively. After 30 min of incubation at room temperature, samples were added on top of the Cushion Buffer containing Taxol and centrifuged at 100,000×*g* for 40 min at room temperature. The pellet (P) and supernatant (S) fractions were collected and separated by SDS-PAGE. The blots were then stained with Ponceau S followed by Western blotting with the anti-HA antibody or with Coomassie brilliant blue (MAP2 and BSA controls).

### Confocal Immunofluorescence Microscopy

Cells grown on coverslips were fixed in 4% paraformaldehyde in PBS for 15 min at room temperature and then washed three times in PBS carefully. Cells were permeabilized in PBS containing 0.25% Triton X-100 (PBST) for 10 min followed by blocking for 30 min in PBS containing 1% BSA at room temperature. Coverslips were incubated for 2 h with anti-β-tubulin and/or anti-EGFR antibodies at room temperature and then washed three times in PBST. Samples were incubated with the secondary antibodies Rhodamine (TRITC)-conjugated goat anti-mouse immunoglobulin G (Jackson) and/or DyLight 594 AffiniPure goat anti-rabbit immunoglobulin G (Earthox) for 1 h at room temperature and then washed three times in PBST. After incubation with 5 μg/ml Hoechst 33258 (sigma) for 5 min at room temperature, coverslips were mounted in ProLong Gold antifade reagent (Invitrogen). Confocal microscopy images were acquired using an Olympus FluoView FV1000 confocal laser scanning microscope (Olympus, Tokyo, Japan) equipped with an oil-immersion Plan apochromat 1.4-NA ×100 objective lens.

### Cell Proliferation Assay

SMMC7721 cells were seeded at 2×10^3^ cells per well in 96-well plates and transfected with HA-TBC1D3, HA-TBC1D3(320∼353) and control HA vector, respectively. After the indicated times, complete medium was replaced with 90 μl of fresh serum-free DMEM medium and 10 μl of Cell Counting Kit-8 (CCK-8, Dojindo Laboratories, Kumamoto, Japan) was added to each well. After incubation in the dark at 37°C for 1 hour, the absorbance was measured with excitation at 450 nm (OD450) using the ELISAmate spectrophotometer (MULTISKAN MK3, Thermo Labsystems).

### Statistical Analysis

Results were expressed as mean values ± standard deviation (mean ± SD). One-way analysis of variance (ANOVA) was performed to calculate statistical significance.

## Results

### TBC1D3 interacts with β-tubulin

**Figure 1:**
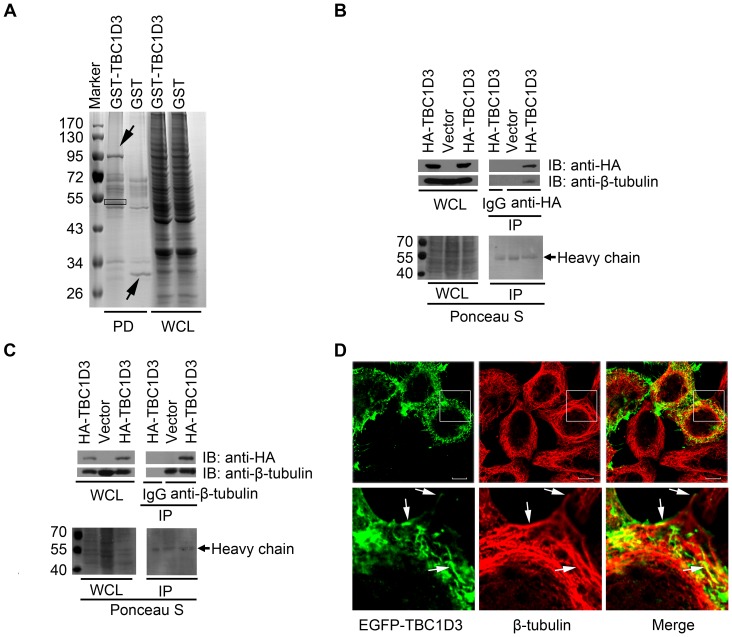
TBC1D3 interacts with β-tubulin. (A) A representative image of the resolved protein eluate following the GST pull-down assay. SMMC7721 cells were transfected with GST-tagged TBC1D3 (GST-TBC1D3) or control GST vector (GST). Cell extracts were pulled down with glutathione-Sepharose 4B beads (PD), washed, resolved by 10% SDS-PAGE and then stained with Coomassie brilliant blue. The bait protein band (GST-TBC1D3 or control GST) and β-tubulin, one of the TBC1D3-interacting proteins identified by mass spectrometry, are indicated by arrows and a rectangle, respectively. Molecular weight markers (Marker) are shown on left in kDa. WCL, whole cell lysate. (B and C) SMMC7721 cells were seeded in three 60-mm dishes. The following day, cells were transfected with HA-tagged TBC1D3 (HA-TBC1D3) (dishes 1 and 3) or control HA vector (Vector) (dish 2). Lysates were immunoprecipitated (IP) with control immunoglobulin G (IgG) (dish 1 in B and C), anti-HA (dishes 2 and 3 in B) or anti-β-tubulin (dishes 2 and 3 in C) antibodies. After SDS PAGE and transfer, the blots were stained with Ponceau S (bottom panels) prior to immunoblotting (IB) with anti-HA and anti-β-tubulin antibodies (top panels). (D) SMMC7721 cells were transfected with EGFP-tagged TBC1D3 (top left, green). Cells were subjected to indirect immunofluorescence with anti-β-tubulin antibody (top central, red), and then analyzed by confocal microscopy. Immunofluorescent images were merged (top right), with yellow revealing overlap (Merge). The indicated areas of the outlined boxes in the top panels are shown in higher magnification in the bottom panels. Arrows indicate co-localizations between TBC1D3 and β-tubulin on filamentous structures. Scale bar, 10 μm.

To gain insights into TBC1D3 function and regulation, we sought to identify its interaction partners using a GST pull-down assay combined with mass spectrometry analysis. SMMC7721 cells were transfected with GST-tagged fusion of human TBC1D3 (GST-TBC1D3) and control GST alone (GST), which were used as bait proteins to screen prey proteins composed of total cellular proteins. When the pattern of protein bands was compared, some bands were unique to the GST-TBC1D3 but not GST interacting assays on Coomassie brilliant blue-stained gel (Figure 1A). The differentially bound protein bands were analyzed by using a MALDI-TOF mass spectrometer and several interacting proteins were identified (Data not shown). Among them, one protein (about 55 kDa, indicated by a rectangle in Figure 1A) matched best with a variety of mammalian β-tubulin isoforms (Table 1), which are multifunctional cytoskeletal proteins. We decided to investigate β-tubulin in this study because of its potential connection with EGFR signaling as described in the discussion section.

**Table 1:** Identification of the TBC1D3-interacting protein with molecular weight of about 55 kDa.

	Accession	Mass	Score	Description	Queries matched
1.	gi|57209813	47736	250	tubulin, beta polypeptide [Homo sapiens]	23
2.	gi|7106439	49639	247	tubulin, beta 5 [Mus musculus]	23
3.	gi|18088719	49640	247	Tubulin, beta [Homo sapiens]	23
4.	gi|2119276	48848	238	beta-tubulin - human (fragment)	22
5.	gi|338695	49727	237	beta-tubulin	22
6.	gi|55731354	41715	235	hypothetical protein [Pongo pygmaeus]	21
7.	gi|5174735	49799	218	tubulin, beta, 2 [Homo sapiens]	20
8.	gi|23958133	49808	218	Tubulin, beta 2C [Homo sapiens]	20
9.	gi|20809886	49776	218	Tubulin, beta 2C [Homo sapiens]	20
10.	gi|27368062	49721	218	class IVb beta tubulin [Homo sapiens]	20

As an independent means of confirming this association, we performed the co-immunoprecipitation with anti-HA antibody. As seen in Figure 1B, endogenous β-tubulin was not co-immunoprecipitated with control immunoglobulin G (IgG) in SMMC7721 cells transiently transfected with HA-TBC1D3, but did exist in anti-HA immunoprecipitates from HA-TBC1D3 but not control vector-expressing cells. Also, the reciprocal immunoprecipitation was performed. TBC1D3 co-immunoprecipitated with endogenous β-tubulin but not control IgG in SMMC7721 cells (Figure 1C). Collectively, these data reveal that β-tubulin is a potential interaction partner for TBC1D3.

As an alternative approach to determine if TBC1D3 interacted with β-tubulin, their subcellular localization was analyzed by confocal microscopy. Since anti-TBC1D3 antibody is not commercially available, EGFP-fusion version of TBC1D3 was produced and then transiently transfected into SMMC7721 cells. An indirect immunofluorescence with anti-β-tubulin antibody was performed. As seen in Figure 1D (left panel), TBC1D3 exhibited localization at the plasma membrane, on filamentous structures, and punctate structures that were often aligned along the filaments. On the other hand, β-tubulin was stained in a radial or reticular array in the cytoplasm (middle panel in Figure 1D). There are partial co-localization between TBC1D3 and β-tubulin/microtubule on the filamentous structures (right panel in Figure 1D). This co-localization pattern suggested at least two possibilities concerning the relationship between TBC1D3 and β-tubulin/microtubule. One possibility is that TBC1D3 indirectly interacts with β–tubulin/microtubule via other proteins such as MAPs. This indirect interaction pattern would lead to co-localization or a lack of co-localization between TBC1D3 and β–tubulin/microtubule in the presence or absence of MAPs, respectively. Another possibility is that localization of TBC1D3 at the plasma membrane is, at least partly, independent of its interaction with β–tubulin/microtubule. Notably, Figure 1D showed that substantial amount of TBC1D3 lacks co-localization with β–tubulin/microtubule at the plasma membrane.

### TBC1D3 associates with α-tubulin and microtubules

**Figure 2:**
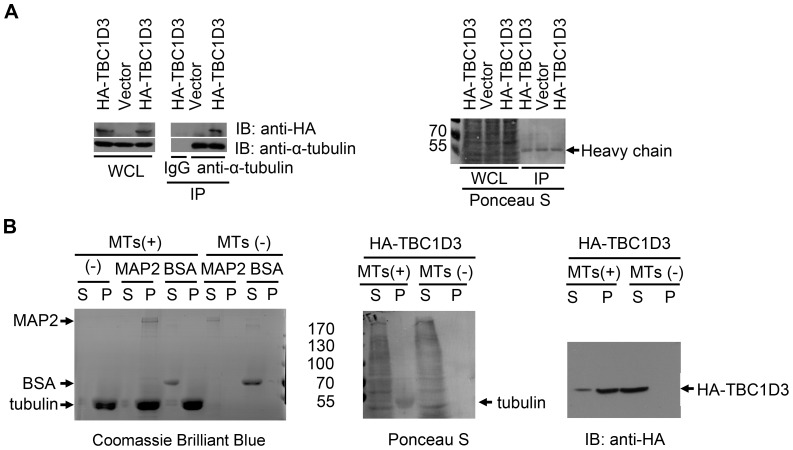
TBC1D3 associates with α-tubulin and microtubules. (A) SMMC7721 cells were seeded in three 60-mm dishes. The next day cells were transfected with HA-TBC1D3 (dishes 1 and 3) or control HA vector (dish 2). Lysates were immunoprecipitated (IP) with control IgG (dish 1), or anti-α-tubulin (dishes 2 and 3) antibodies. After SDS PAGE and transfer, the blots were stained with Ponceau S (right panel) prior to immunoblotting (IB) with anti-HA and anti-α-tubulin antibodies (left panel). Molecular weight markers are shown on left in kDa. WCL, whole cell lysate. (B) SMMC7721 cells were transfected with HA-TBC1D3 and lysed in General Tubulin Buffer (see details in Materials and Methods). Cell lysates were subjected to ultracentrifugation to remove polymerized microtubules. Clarified lysates were then added to the Taxol supplemented General Tubulin Buffer alone (MTs(-)) or together with polymerized microtubules (MTs(+)). After incubation at room temperature, samples were placed on top of the Cushion buffer containing Taxol and centrifuged at 100,000×*g*. The pellet (P) and supernatant (S) fractions were collected and separated by SDS PAGE. The blots were then stained with Ponceau S (middle panel) followed by immunoblotting (IB) with anti-HA antibody (right panel). Positive (MAP2) and negative controls (BSA and no protein (-)) were also performed and detected by Coomassie brilliant blue staining (left panel).

α- and β-tubulin form heterodimers and assemble dynamic microtubules, which are involved in maintenance of cell structure and transport of intracellular vesicles and organelles as well as cellular signal transduction. Since TBC1D3 associated with β-tubulin, we examined whether it associates with α-tubulin. To test this, we performed the co-immunoprecipitation with anti-α-tubulin antibody. As shown in Figure 2A, HA-TBC1D3 was not present in anti-α-tubulin immunoprecipitates from control vector-expressing cells. In contrast, TBC1D3 did co-immunoprecipitate with endogenous α-tubulin but not control IgG in SMMC7721 cells transiently transfected with HA-TBC1D3 (Figure 2A), indicating that TBC1D3 associates with α-tubulin.

We next performed microtubule cosedimentation assays using polymerized microtubules. As shown in Figure 2B (right panel), TBC1D3 bound to and was co-sedimented with the microtubules, while TBC1D3 alone did not pellet. In control sedimentation assay (Figure 2B, left panel), MAP2 bound to microtubules, but BSA did not. Neither of the two proteins pelleted in the absence of microtubules. In aggregate, these results suggest that TBC1D3 associates with tubulin dimer/oligomer and microtubules.

### Mapping of the β-tubulin-interacting site in TBC1D3

**Figure 3:**
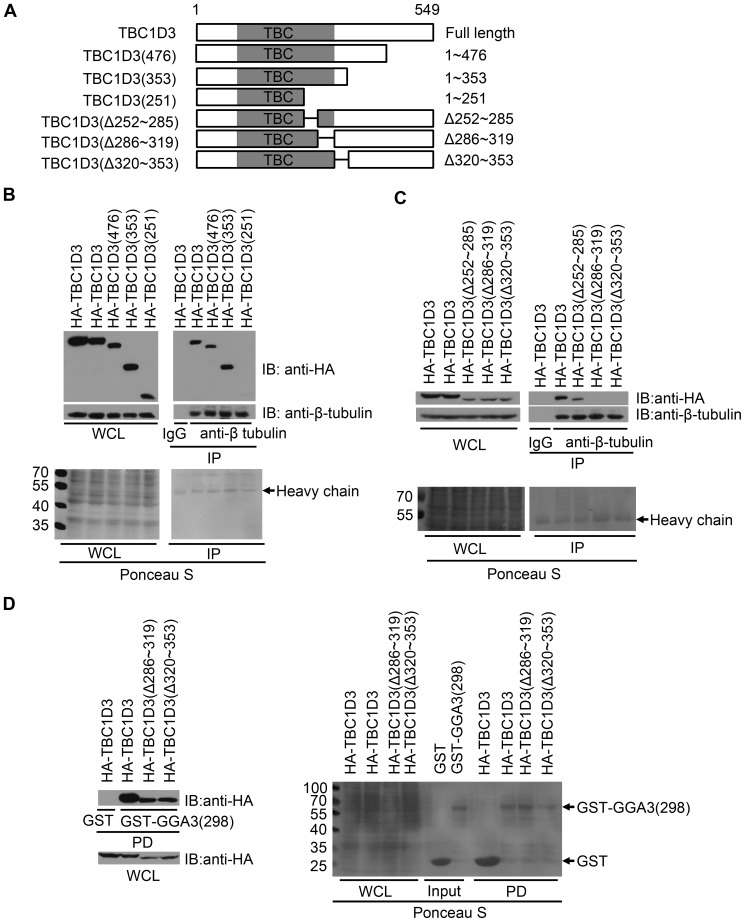
Mapping of β-tubulin-interacting site in TBC1D3. (A) Schematic representation of full-length TBC1D3 and various deletion mutants. The length or internal deletion (Δ) of each isoform in amino acids is indicated. TBC/Rab GAP homology domain is shown in gray. (B and C) SMMC7721 cells were transfected with HA-tagged TBC1D3 and mutants encoding the indicated N-terminal residues of TBC1D3 (B) or harboring internal deletions of the indicated residues (C). Lysates were immunoprecipitated (IP) with anti-β-tubulin antibody or control IgG. After SDS PAGE, the blots were stained with Ponceau S (bottom panels) followed by immunoblotting (IB) with anti-HA and anti-β-tubulin antibodies (top panels). Molecular weight markers are shown on left in kDa. WCL, whole cell lysate. (D) SMMC7721 cells were seeded in four 60-mm dishes. The following day, cells wre transfected with HA-tagged TBC1D3 (dishes 1 and 2) or mutants harboring internal deletions of the indicated residues (dishes 3 and 4). Cell extracts were pulled down (PD) with bacterially purified GST-GGA3(298) (dishes 2, 3 and 4) or GST (dish 1) immobilized on glutathione-Sepharose 4B beads. After SDS PAGE, the blots were stained with Ponceau S (right panel) followed by immunoblotting (IB) with anti-HA antibody (left panels).

To determine the β-tubulin-interacting site of TBC1D3, a series of nested deletion mutants were generated, which encodes the indicated N-terminal residues of TBC1D3 (Figure 3A), and transfected into SMMC7721 cells. Co-immunoprecipitations with anti-β-tubulin antibody or control IgG were performed, and association of TBC1D3 mutants was then immunoblotted with anti-HA and anti-β-tubulin antibodies. As a negative control, HA-TBC1D3 did not bind to endogenous β-tubulin in the presence of IgG (Figure 3B). TBC1D3(476) and TBC1D3(353) mutants bound strongly to β-tubulin in the presence of anti-β-tubulin antibody as efficiently as full-length of TBC1D3 (Figure 3B). In contrast, a construct containing the first 251 amino acids (TBC1D3(251)) failed to associate with β-tubulin (Figure 3B). These data suggest that the β-tubulin-interacting site resides at or near amino acids 252∼353, which comprises the C-terminus of TBC/Rab GAP homology domain (Figure 3A).

To pinpoint the site of interaction, internal deletion mutants were generated (Figure 3A). As shown in Figure 3C, an internal deletion of amino acids 252∼285 had no effect on interaction between TBC1D3 and endogenous β-tubulin while deletion of amino acids 286∼319 or 320∼353 abolished interaction of TBC1D3. To ensure that both of the internal mutants were well folded and functional, we confirmed their ability to associate with a GST-tagged C-terminal truncation mutant encoding the first 298 amino acids of GGA3, which contains the known TBC1D3-binding site, as efficiently as wild-type TBC1D3 (Figure 3D). In contrast, as a negative control, wild-type TBC1D3 did not bind to GST protein (Figure 3D). Taken together, these results indicate that optimal interaction between TBC1D3 and β-tubulin requires amino acids 286∼353 near the C-terminus of the TBC1D3 domain.

### The subcellular distribution of TBC1D3 is regulated by microtubules

**Figure 4:**
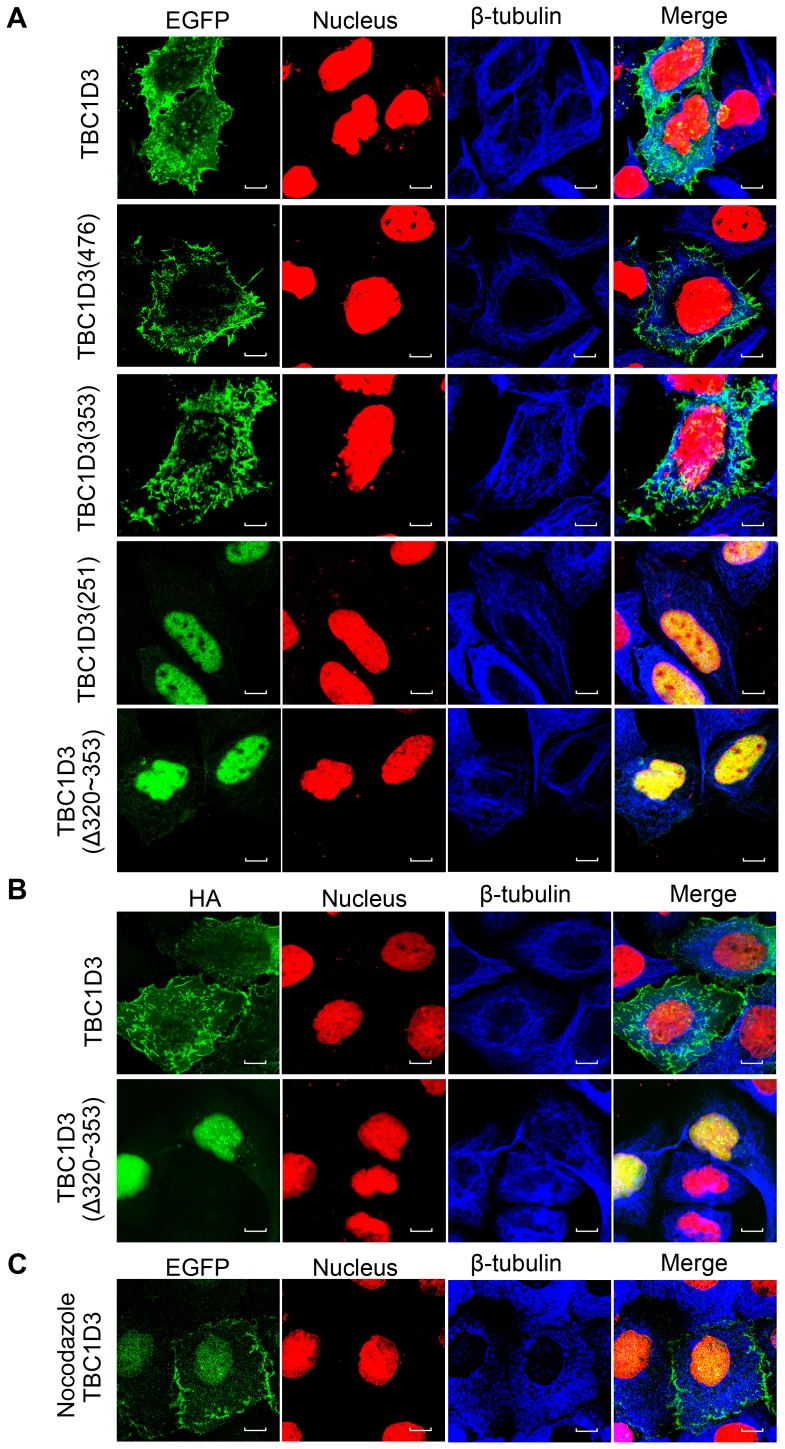
The subcellular distribution of TBC1D3 is regulated by the microtubule network. SMMC7721 cells were transfected with EGFP (A and C, green) or HA (B) -tagged TBC1D3 and mutants encoding the indicated N-terminal residues of TBC1D3 or harboring internal deletions of the indicated residues. EGFP-tagged TBC1D3-transfected SMMC7721 cells were treated with nocodazole (40 μg/ml) for 2 h (C). Cells were subjected to indirect immunofluorescence with anti-β-tubulin antibody (blue) alone (A and C), or together with anti-HA antibody (B, green), stained with Hoechst 33258 (red), and then analyzed by confocal microscopy. Immunofluorescent images were merged with yellow revealing overlap (Merge). Scale bar, 10 μm.

Microtubules provide a spatially extensive docking platform for many signal molecules and spatially regulate signal transduction by at least three distinct mechanisms: MT sequestering and release, MT delivery and MT scaffolding of the molecules [29], [30]. Since TBC1D3 associates with tubulin/microtubules, it was unclear whether interaction with tubulin/microtubule might be required for TBC1D3's subcellular distribution. To address this issue, we generated EGFP-fusion version of TBC1D3 and mutants encoding the indicated N-terminal residues of TBC1D3 or harboring internal deletions of the indicated residues, then transiently transfected SMMC7721 cells with these constructs, and analyzed their localization using confocal microscopy. Similar to wild-type TBC1D3, the deletion mutants TBC1D3(476) and TBC1D3(353) exhibited localization at the plasma membrane, on cytoplasmic filamentous and punctate structures (Figure 4A). In contrast, TBC1D3(251) and TBC1D3(Δ320-353), which have no β-tubulin-interacting site, appeared mainly in the nucleus instead of cytoplasmic filamentous and punctate structures although their plasma membrane localization persisted (Figure 4A). Since EGFP-fusion proteins are often used in many studies, it was unclear whether nuclear localization of the TBC1D3 mutants was not based from the nature of EGFP fusion. To explore this issue, we tested HA-tagged TBC1D3 and mutants for their distribution in SMMC7721 cells. As seen in Figure 4B, HA fusion variants exhibited the same distribution as their respective EGFP-fusion constructs. These data suggest that association with tubulin/microtubule is required for cytoplasmic filamentous and punctate localization but not for plasma membrane localization of TBC1D3.

Since TBC1D3 associates with microtubules, we next examined whether disruption of microtubules affected wild-type TBC1D3 distribution. In the presence of the microtubule depolymerizing drug nocodazole, cytoplasmic TBC1D3-positive filaments and punctates were completely abolished and wild-type TBC1D3 instead appeared in the nucleus (Figure 4C). In contrast, plasma membrane localization of TBC1D3 persisted (Figure 4C). Unlike nocodazole, EGF stimulation had no effect on wild-type TBC1D3 distribution (Data not shown). Together, these results indicate that the localization of TBC1D3 is spatially regulated by microtubules. Without the microtubule-mediated sequestration, TBC1D3 translocates from the cytoplasm into the cell nucleus.

### Microtubules are involved in TBC1D3-mediated regulation of EGFR ubiquitination

**Figure 5:**
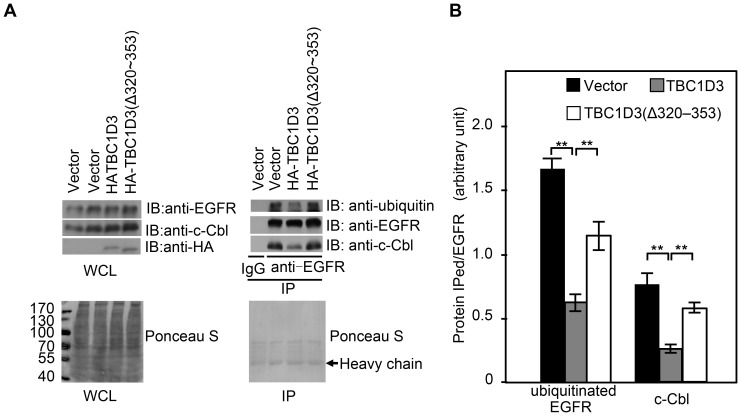
Microtubules are involved in TBC1D3-mediated regulation of EGFR ubiquitination. (A) SMMC7721 cells were seeded in four 60-mm dishes. The following day, cells were transfected with control HA vector (dishes 1 and 2), HA-TBC1D3 (dish 3) and HA-TBC1D3(Δ320–353) (dish 4), respectively. The next day cells were serum-starved and then treated with 100 ng/ml of EGF for 15 minutes. Lysates were immunoprecipitated (IP) with anti-EGFR antibody (dishes 2, 3 and 4) or control IgG (dish 1). After SDS PAGE, the blots were stained with Ponceau S (bottom panels) followied by immunoblotting (IB) with anti-c-Cbl, anti-EGFR, anti-HA and anti-ubiquitin antibodies (top panels). Anti-ubiquitin and anti-HA antibodies were used to monitor ubiquitinated EGFR, HA-TBC1D3 and its mutant. Molecular weight markers are shown on left in kDa. WCL, whole cell lysate. (B) The bar graph is derived from densitometric analysis of Western blots as typified in top right panel in A. Ubiqitinated EGFR and c-Cbl are normalized to EGFR total amounts. The data are presented as means ± SD of three independent experiments (**, p<0.01).

Ubiquitination of EGFR plays a critical role in protection against excessive activation of the activated receptor. EGFR is ubiquitinated by c-Cbl, an E3 ligase, both at the plasma membrane and in the cytoplasmic endosomes [9]. TBC1D3 inhibits the recruitment of c-Cbl and ubiquitination of EGFR [26]. However, the localization where TBC1D3 inhibits the c-Cbl-mediated ubiquitination was not examined. It was also unknown whether microtubules are involved in TBC1D3-mediated suppression of the c-Cbl recruitment and EGFR ubiquitination. To address these issues, we examined the c-Cbl recruitment and EGFR ubiquitination in SMMC7721 cells expressing control vector, wild-type TBC1D3, and β-tubulin interacting-deficient TBC1D3 mutants which disappeared from cytoplasmic filamentous and punctate structures but not plasma membrane. As seen in Figure 5, TBC1D3 expression substantially reduced the c-Cbl recruitment and EGFR ubiquitination compared to control vector. In contrast, the TBC1D3 mutants lost the inhibitory effect (Figure 5). As a negative control, endogenous EGFR and c-Cbl were not co-immunoprecipitated with control IgG in SMMC7721 cells transiently transfected with control vector (Figure 5, top right panel). Together, these data indicate that association with microtubules is required for TBC1D3 to suppress the c-Cbl recruitment and EGFR ubiquitination. Since the cytoplasmic TBC1D3-positive punctate and filamentous structures may represent endosomal and tubular endosomal compartments, respectively, these results also suggest that TBC1D3 inhibits the c-Cbl-mediated EGFR ubiquitination in the cytoplasmic endosomes, not at the plasma membrane.

### Microtubule network modulates the enhancement of EGFR stability and activation of EGFR signaling by TBC1D3

**Figure 6:**
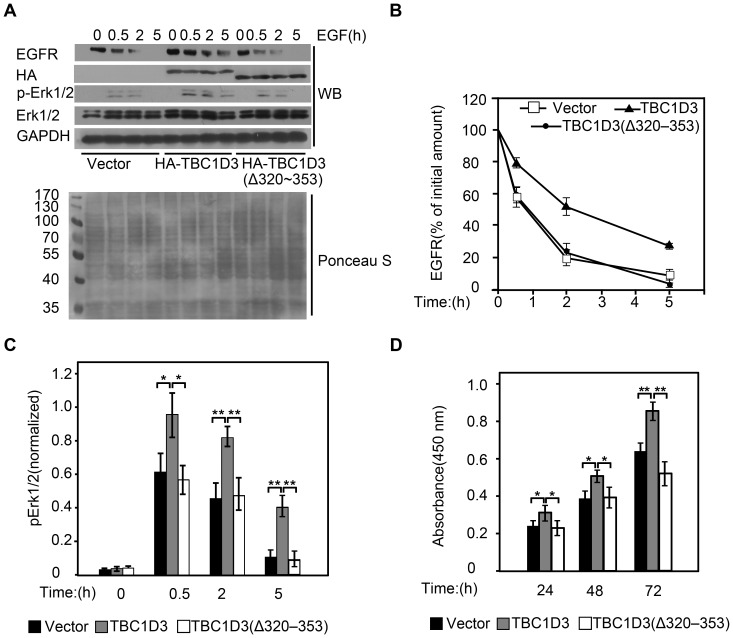
Microtubule network modulates the enhancement of EGFR stability and activation of EGFR signaling by TBC1D3. (A) SMMC7721 cells were transfected with HA-TBC1D3, HA-TBC1D3(Δ320–353) and control HA vector, respectively. After 24 h, cells were serum-starved, pretreated with cycloheximide (40 μg/ml) for 2 h, and then stimulated with 100 ng/ml of EGF for the indicted times. After cell lysates were resolved by SDS-PAGE, the blot was stained with Ponceau S (bottom panel) followed by Western blotting (WB) with anti-EGFR, anti-Erk1/2, anti-phospho-Erk1/2 (p-Erk1/2), anti-HA and anti-GAPDH antibodies (top panel). Molecular weight markers are shown on left in kDa. The line (B) and bar (C) graphs are derived from densitometric analysis of Western blots as typified in top panel in A. The percentage of the amount of EGFR present at the indicated time of EGF stimulation over the initial amount in each group was quantified in B. p-Erk1/2 is normalized to Erk1/2 total amounts in C. The data are presented as means ± SD of three independent experiments (*, p<0.05; **,p<0.01). (D) SMMC7721 cells were transfected with HA-TBC1D3, HA-TBC1D3(Δ320–353) and control HA vector, respectively. After incubation in 10% FBS-DMEM for the indicated times, their survival was determined by the CCK8 assay. Cell growth rate is expressed as absorbance at 450 nm. Data are presented as Means ± SD of sextuplicates from one experiment representative of three performed (*, p<0.05; **, p<0.01).

Having determined that association with microtubules is essential for TBC1D3 to suppress EGFR ubiquitination, we next examined role of microtubules in TBC1D3-medicated enhancement of EGFR stability and signaling. As seen in Figure 6A and 6B, cells transfected with control vector showed a rapid degradation of EGFR; after 30 minutes of EGF stimulation, over 40% of the EGFR signal was lost, and only about 20% of EGFR proteins were left after 2 hours. In contrast, EGFR degradation was significantly delayed in cells expressing TBC1D3; merely 20% of the receptor population was degraded after 30 min, and more than 50% of EGFR proteins persisted after 2 hours (Figure 6A and 6B). However, deficiency in β-tubulin interacting resulted in TBC1D3's inability to delay EGFR degradation; about 40% and over 75% of the EGFR signal were lost after 30 min and 2 hours, respectively (Figure 6A and 6B). These results indicate that association with microtubule is required for regulation of EGFR stability by TBC1D3.

Phosphorylation of Erk1/2 on T202 and Y204 is a well-established downstream marker for activation of EGFR signaling. Since microtubule involved in TBC1D3-enhanced EGFR stability, we next examined phosphorylation level of Erk1/2 in SMMC7721 cells transfected with control vector, HA-TBC1D3 and β-tubulin interacting-deficient TBC1D3 mutants. Total cellular contents of Erk1/2 were used to normalize the results in each sample (Figure 6A). As seen in Figure 6A and 6C, following EGF stimulation for 30 minutes phosphorylation levels of Erk1/2 increased in all of the transfected cells. The EGF-induced Erk1/2 activation was enhanced by expression of TBC1D3, but not TBC1D3 mutants deficient in β-tubulin interacting (Figure 6A and 6C). Subsequently, the phosphorylation levels of Erk1/2 decreased and Erk1/2 signaling in both control vector and the TBC1D3 mutant-transfected cells subsided after 5 hours, whereas TBC1D3-expressing cells sustained their Erk1/2 activation during this period (Figure 6A and 6C). Consistent with these results, TBC1D3 expression stimulated proliferation of SMMC7721 cells, but TBC1D3 mutant deficient in β-tubulin interacting had no such effect (Figure 6D). Taken together, these results indicate that association with microtubule is required for TBC1D3 to regulate EGFR stability and signaling.

## Discussion

Our studies identify tubulin/microtubule network as a novel regulator of TBC1D3. Interaction of β-tubulin is mediated by amino acids 286∼353 within TBC1D3. Deletion mutations in these amino acids significantly attenuate interaction with β-tubulin. Furthermore, through combined analysis of β-tubulin interacting-deficient TBC1D3 mutants and the pharmacological agent nocodazole that interferes with the polymerization of microtubules, we identify TBC1D3 as a novel nucleocytoplasmic protein. Inability to bind β-tubulin and disruption of the microtubule network result in disappearance of TBC1D3 from cytoplasmic filamentous and punctate structures and TBC1D3 instead transports into the nucleus. Finally, our results indicate that the cytoplasmic retention of TBC1D3 by the microtubule network is required for enhanced EGFR stability and signaling.

The precise mechanism by which β-tubulin/microtubule network modulates the activation of EGFR signaling by TBC1D3 remains to be determined. The fact that TBC1D3 mutants deficient in β-tubulin interacting had no ability to delay EGFR degradation and to enhance EGFR signaling indicates that interaction between TBC1D3 and β-tubulin is required. Two possible mechanisms are by regulating inhibitory impact of TBC1D3 on microtubule acetylation or EGFR ubiquitination. Microtubule acetylation is under the control of balanced tubulin acetyltransferases/deacetylases. Acetylatransferases (such as ELP3 and ARD1) and deacetylases (including HDAC6 and SIRT2) associate with microtubules and function to acetylate and deacetylate α-tubulin, respectively [31]. Before EGF stimulation, EGFR associates with HDAC6, which inhibits microtubule acetylation. Upon EGF binding, EGFR phosphorylates and deactivates HDAC6, thereby promoting microtubule acetylation [32], which increases the affinity and processivity of microtubule motors [33], [34], and results in accelerated segregation of EGFR from early endosomes and premature delivery of EGFR to the late endosomal and lysosomal compartments for degradation [35]. Also, c-Cbl and Cbl-b interact with β-tubulin and promote microtubule acetylation by displacing HDAC6 from β-tubulin [36]. However, we observed that TBC1D3 had no impact on β-tubulin binding to both HDAC6 and c-Cbl (data not shown), and microtubule acetylation (Figure S1), demonstrating the former mechanism is unlikely. Nevertheless, it remains possible that TBC1D3 inhibits directly the interaction of microtubule with its motors including dynein and kinesin 1 in a microtubule acetylation-independent manner. Functional interference with motor proteins or cellular trafficking systems will contribute to clarification of the function.

It appears more likely that β-tubulin interaction promotes TBC1D3-enhanced EGFR signaling by regulating TBC1D3's ability to inhibit EGFR ubiquitination. Intriguingly, TBC1D3 mutants deficient in β-tubulin interacting not only lost the ability to inhibit c-Cbl recruitment and EGFR ubiquitination, but also disappeared from the cytoplasm although plasma membrane localization of TBC1D3 persisted. These results touch upon the poorly understood issue of where TBC1D3 inhibits EGFR ubiquitination [26]. It has been speculated that c-Cbl ubiquitinates EGFR both at the plasma membrane and in the cytoplasmic endosomes [9]. Although sufficient for EGFR internalization, EGFR ubiquitination at the plasma membrane is not essential for the process [11]–[13]. EGFR ubiquitination in the endosomes, however, is indeed required for its sorting onto intraluminal vesicles of multivesicular endosomes/bodies and subsequent lysosomes for efficient degradation [12], [14]. Notably, the TBC1D3 mutants had no ability to enhance EGFR stability and signaling, suggesting that TBC1D3 may function in the cytoplasmic endosomes, not at the plasma membrane, to inhibit c-Cbl recruitment and EGFR ubiquitination. Consistent with this notion, EGFR internalization was not inhibited, but enhanced, by TBC1D3 [26], although these results might be explained by increased expression and recycling of EGFR in TBC1D3-expressing cells. In addition, there are at least two deubiquitinating enzymes including AMSH [37], [38] and Cezanne-1 [39], which remove ubiquitin from EGFR in endosomes. It is possible that TBC1D3 functions in the cytoplasmic endosomes to stimulate the action of the enzymes and prevent ubiqutin-mediated degradation of EGFR.

In this study, we identify TBC1D3 as a novel nucleocytoplasmic protein that is retained by the microtubule network in the cytoplasm. It has been speculated that there exist two opposing modes of microtubule-regulated nucleocytoplasmic transport, including microtubule-facilitated and -inhibited nuclear import [40]. In the first mode, the microtubule network functions as tracks of the motor protein dynein to promote the nuclear translocation of proteins such as hypoxia inducible factor-1, p53, parathyroid hormone-related protein, retinoblastoma protein, and the rabies virus P-protein [41]–[48]. Dynein transports various cellular cargoes on microtubules towards the minus-end of the microtubule, which is usually oriented from the cell periphery to the nucleus [49]. The microtubule network and dynein, therefore, are thought to facilitate nuclear import [44], [46], [48]. In contrast, the microtubule network have also been implicated in binding and subsequently sequestering at least three nucleocytoplasmic proteins, c-myc, MIZ-1 and SRY-related HMG box-9 (SOX9), in the cytoplasm, thereby inhibiting their nuclear import and resulting in suppression of their transcriptional activity [41], [50]–[53]. At variance with these proteins, which function in the nucleus as transcriptional factors, we observed that deficiency in β-tubulin interacting or disruption of the microtubule network caused not only disappearance of TBC1D3 from the cytoplasm but also inability to enhance EGFR signaling, demonstrating that TBC1D3 is a novel nucleocytoplasmic protein that functions in the cytoplasm retained by the microtubule network. However, we cannot exclude the possibility that TBC1D3 also functions in the nucleus, which may provide novel insights into its function in that case.

## Supporting Information

Figure S1 **TBC1D3 does not affect the acetylation of α-tubulin.** SMMC7721 cells were transfected with HA-TBC1D3 and control HA vector, respectively. After 24 h, cells were serum-starved and then stimulated with 100 ng/ml of EGF for the indicted times. Lysates were immunoblotted with anti-c-Cbl, anti-HDAC6, anti-HA, anti-acetyl-α-tubulin and anti-GAPDH antibodies.
(TIF)
