# Supplementary figures and images for: Cytoplasmic Retention of a Nucleocytoplasmic Protein TBC1D3 by Microtubule Network Is Required for Enhanced EGFR Signaling
Source: PLoS One. 2014 Apr 8;9(4):e94134. doi: 10.1371/journal.pone.0094134 (PMC3979746; doi:10.1371/journal.pone.0094134)

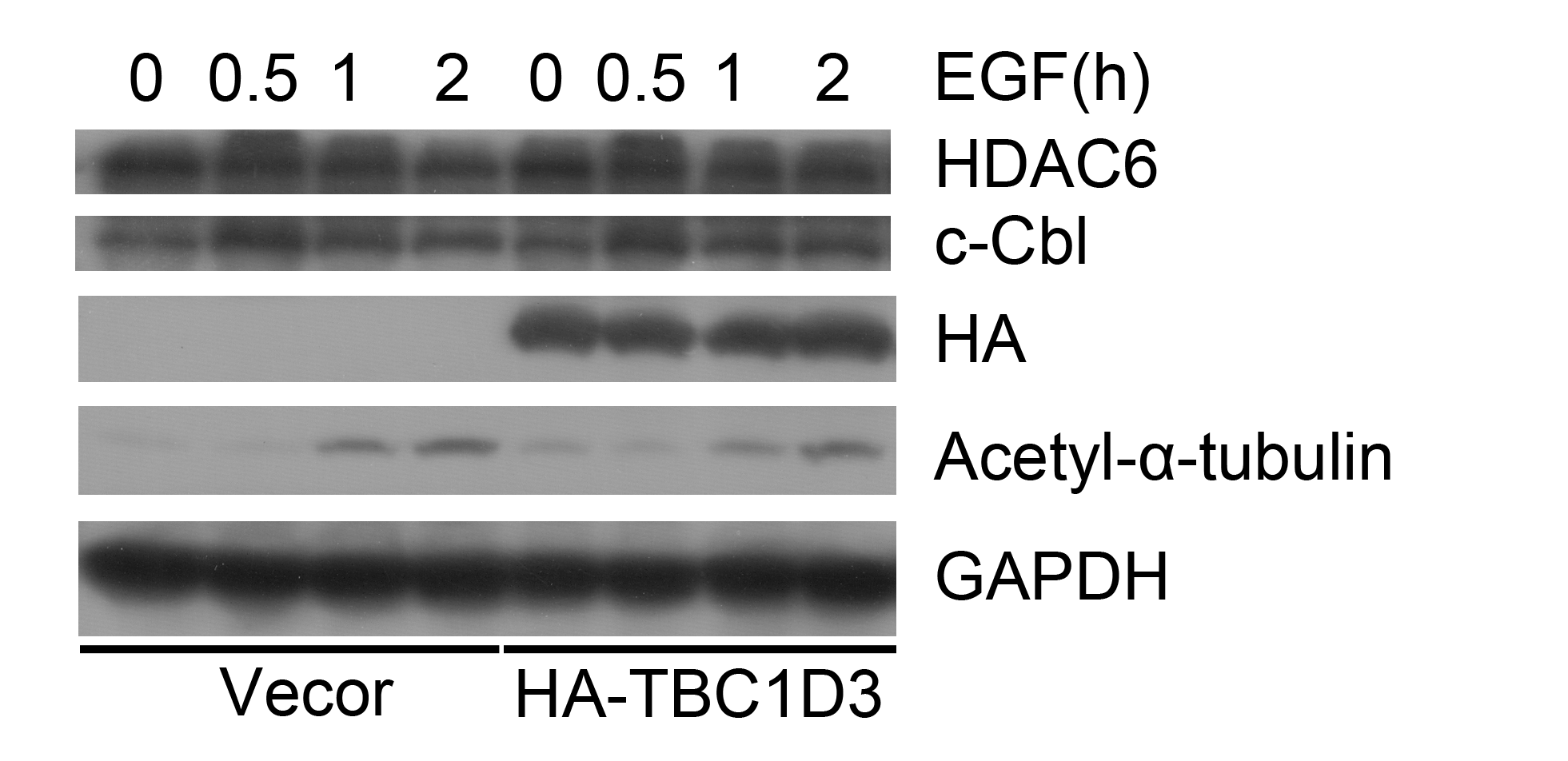

Supplement: Figure S1 — TBC1D3 does not affect the acetylation of α-tubulin. SMMC7721 cells were transfected with HA-TBC1D3 and control HA vector, respectively. After 24 h, cells were serum-starved and then stimulated with 100 ng/ml of EGF for the indicted times. Lysates were immunoblotted with anti-c-Cbl, anti-HDAC6, anti-HA, anti-acetyl-α-tubulin and anti-GAPDH antibodies. (TIF) [file pone.0094134.s001.tif]
